# Proceedings of Maryland and District of Columbia Dental Association. Annual Meeting

**Published:** 1881-02

**Authors:** 


### AMERICAN JOURNAL
OF
### DENTAL SCIENCE.

**Vol. XIV. THIRD SERIES - FEBRUARY, 1881. No. 10.**

### ARTICLE I.

*Proceedings of Maryland and District of Columbia Dental Association.—Annual Meeting.*—(Continued.)

Discussion being in order, Dr. Winder took the floor and said :—

Mr. President, this Association can only feel highly gratified by such a paper, and is forced to indorse the truths contained in it. This mode of filling prevents, in mastication, food getting between the teeth. It not only does that, but it certainly keeps the necks of the teeth apart, allowing saliva to pass through and wash thoroughly. The only criticism I can possibly make is, that probably the form of the tooth has more to do with its protection than the structure itself. I believe that a set of teeth up to the morphological law will stand better than a set of teeth not so well formed but of better quality

We find them almost a plain surface from the cervical to the cutting edge; the plain flat surfaces coming in contact with each other.

I do not know what Dr. Webb would do in this case. If all teeth were up to the highest condition, I think there would be but one opinion about this matter. Of course, in the study of the preparation of the mouth for the final end, the object naturally would be to bring that mouth as near the high type as is possible to do it. But it is very often almost impossible to bring a mouth up to the highest type. I have personally to thank Dr. Webb for the paper. I think it one that we should be very glad to accept, because in my opinion it represents the most advanced progress in the mode of filling teeth.

Mr. President.—I would just ask Dr. Webb to make a statement in regard to the question Dr. Winder asks.

Dr. Webb.—I would there restore also, and make the margins free. I would more than restore, if necessary, to make the margins free.

Dr. Donaldson.—I would much rather, this evening, hear other gentlemen express opinions upon this subject than to attempt to discuss it myself. I fully admit what Dr. Winder has stated in regard to the "highest type" of teeth but, as he says, we rarely meet with these highest types of teeth where the necks are separated considerably, while the crowns touch at a mathematical point. If all the cases met with in our practice were of that kind, there would be no diversity of opinion as to the correctness of the practice advocated by Dr. Webb; but I must differ with him when he states that his mode of practice is best in *all cases*, because my own experience satisfies me that such is not the case. I do not like the plan suggested by Dr. Webb of enlarging the whole or a part of the arch, and keeping the neck of the teeth apart by contouring when they were not so naturally. In cases where the molars, bicuspids and canines all come together from the masticating surface to the gum—and are all decayed upon the approximal surfaces, and we often meet with such cases, by pursuing this plan of operation the whole arch would be considerably enlarged, and in many cases the articulation of the teeth would be interfered with, and consequent discomfort to the patients would follow.

There are other objections to my mind, to this practice, one of which is the inability of so many of our patients to pay for such operations, to say nothing of their unwillingness to bear the fatigue, pain and discomfort consequent upon the performance of them.

We all contour teeth with gold occasionally, and theoretically, the system advocated by Dr. Webb, would seem to be correct, but practically it must be departed from in a great many instances if we would preserve the teeth of many of those who claim our services. In my own pracice I have used, and continue to use cohesive gold in some cases, and sometimes I contour teeth, but most of my filling operations are performed with non-cohesive foil (Abbey's soft,) and my experience is that I can best preserve the greater portion of the teeth I meet with in my practice by using this form of gold.

Dr. Webb.—I take it in many of these cases where teeth come flatly in contact they are of first class structure. I do not mean that the arch should be enlarged. If we go beyond the margin of the gum, we find that the tooth narrows down. If the cavity be prepared above the margin of the gum, we find that there will be space at the neck, and the margin can be made free. This is on theory. It is the result of close, careful observation; it is the result of practice in a place of 25 or 30 thousand.

If time, opportunity or means are not afforded for such operations the teeth should be kept in condition with soft fillings until time does present itself.

If cohesive gold is properly manipulated, I do know that it can be placed better than soft foil.

Dr. Donaldson.—How many children of the people who are in ordinary circumstances would preserve their teeth until they grew up, if they had to be filled with cohesive gold and built out? I like to see work such as I have seen of Dr. Webb's; I admire the skill he displays; I admire his interest in the profession which brings him to dental meetings throughout the country, and incites him to prepare such papers as we listened to to-night, but at the same time I am forced conscientiously to differ with him in some of his positions.

My own practice is exclusively amongst families that I attend to year after year. I have the teeth of those families in my charge. I could not "contour" all the teeth that come to me needing to be filled, and do my duty. I do not want the members of this Association to look upon me as one standing in the way of true practice in my profession, but these things I say conscientiously, believing them to be true.

Dr. Smythe.—There is one thing that Dr. Webb does not take into account, and that is, that there are few men in the United States who have his manipulative ability. I do not say it in flattery. I honestly think there are very few men capable of putting in fillings, such as I have seen put in by Dr. Webb. It would be a nice thing if all men in the profession would do it, but it is impossible to have it so. I take exception to Dr. Webb when he says teeth cannot be separated and saved, because I have seen fillings in the mouth saving the teeth for 30 and 35 years,—separated teeth and soft gold. If we could all work as well as Dr. Webb we would contour as far as our means would allow us.

Dr. Winder.—We all admit that it is much more difficult to perform an operation thoroughly with co-hesive than with non-cohesive gold—at least that is my experience. But you have all met with fillings, so imperfectly packed with so-called soft foil, that you could easily stick an excavation through the filling, and yet year after year these fillings seem to preserve the teeth—In these cases it would seem that the gold had never been well consolidated, and yet the teeth are preserved—this to me has always been a mystery. I agree with Dr. Webb that cohesive gold can be as thoroughly adapted to the cavity walls, but I think it requires more skill and care. The question of contouring or not should always be governed by the morphology of the teeth, and when teeth come up to the highest morphological type they rarely decay.

Dr. Atkinson.—Mr. President and gentlemen, this is a subject of the success of our lives, and, if we understand it thoroughly there will be no difference of opinion more than there is about mathematics. I want to say something about this subject of heat. An admirable instance came to my mind when Prof. Winder spoke of the fillings put in by Dr. Maynard, and they were solid. When we first began to anneal gold for the purpose of drying it, we made it solid without knowing why. Prof. Winder asks why teeth are preserved so long by fillings so soft that an excavator can be put through them?

That is just like a thousand and one mistakes we make by rushing at conclusions.

He assumes that the filling was the preservative of the tooth which I doubt is true.

Repeatedly have I seen teeth excavated and filled, and the others not, where both were being preserved. I remember a case in the days of stage travelling—a lady was in the chair for the purpose of having the central incisors filled. They were prepared, and one filled when the stage drove up, and she could not sit to have the other filled. I saw her well on to 25 years afterwards. The teeth were of a good class, good morphological form, and good arrangement.

Those incisors were worn down, and only one-third of the length of the cavity that was prepared was left—a crescentic shaped cavity, and the one that was filled was worn down gold and all.

At that time the question arose in my mind that seemed to puzzle Prof. Winder, how it was that the tooth not filled was preserved as well as the other, and it was that the protoplasmic cells are calcified.

And this preservative process that we know nothing about is probably, the thing we want. We jump at conclusions too much.

In filling teeth it is not so much the material we use as it is the manner in which it is done. Anyone that understood the paper read to us, need not go a step beyond that; it is as simple as the multiplication table. If those slab-like teeth that are so very hard were decayed, and they have their thick necks, I would fill them so as to knuckle the gold against gold, and then I would not build it quite out at the neck. Now why? So that the festoon of the gum shall have room enough to come down in such mass as to fill that up, and prevent food from getting in there. Now about fissures. If you find a simple cavity in a tooth, or even evidence of decay, no matter how slight, do not trust it; cut out those fissures and fill them religiously with any gold with which you can make a solid filling. If a child's mouth is treated in this way, in a great majority of cases you will never have anything more to do than to keep his teeth clean. I have seen beautiful fillings and signs of calcareous deposits on the teeth. Do you call that finished? Keep the teeth clean, and the souls will be kept clean.

Dr. H. B. Noble then read the following paper on Operative Dentistry:

In the writings and discussions upon filling teeth, the idea seems general, that one must use either cohesive or non-cohesive foil, and each, to the exclusion of the other in each particular case or filling. In the jurisdiction of this Society there are a large number of our best men using both cohesive and non-cohesive foil in the same cavity; yet very little is said about it in discussions at our meetings, and their combined use seldom advocated in our public gatherings, lecture rooms, or in our dental journals; so that the student, or young men scarcely catch the idea, or are instructed in this manner of operating.

I believe that a large number, probably a majority, of cavities can be best filled by the use of both cohesive and non-cohesive foil, placing the soft or non-cohesive foil next to the walls of the cavity, as it can be more easily and certainly brought in perfect contact, as the ribbons, or cylinders of gold slide upon, or into each other with comparatively little force; but if the gold be cohesive, each little particle or piece adheres to its neighbor at irregular points, forming thousands of arches, that have to be broken down, either by blows or pressure, before perfect union or contact can be had. When the walls of the cavity have been covered, and the larger portion of the cavity filled, non-cohesive foil of such shape as may best suit the operator, or cavity, (generally ribbons;) we begin the use of cohesive foil with very small ribbons, and by the use of small points forcing it into the soft foil in every direction, and at all points anchoring it thoroughly into the soft foil, working up any depression until we have an even uniform surface, which can be built to any desired shape or size.

Soft foil acts as a kind of cushion to protect the tooth from the jars of the mallet, which in sensitive or weak teeth may be of great advantage, also lessening the danger of sore, lame teeth, in a long protracted operation.

After reading the paper Dr. Noble said:—

Mr. President, this has been our practice for many years and we are very loathe to give it up entirely. We have not only seen teeth where the fillings were put in with soft foil, but we have also seen such as Dr. Winder mentioned.

I have seen fillings put in by Dr. Maynard 45 years ago preserving the teeth perfectly. I have been using the combination of tooth foils, and I am satisfied in a great many cases (I believe I am not alone) that we can use the soft foil next the walls of the cavity, and still get a hard surface with the cohesive gold. I think gold can be brought more perfectly in contact with the walls of the cavity, if the non-cohesive form is used.

Dr. Webb.—Mr. President, there are several points to which I wish to refer. In the first place we ought not to have any little arches to break down. Gold should be cut into small pieces, and placed where it wants to be when in the cavity, and malleted down. The class of teeth referred to in reference to filling the bottom with non-cohesive gold so as to give a cushion, should have the phosphate of zinc in the bottom instead. In all cases where the cavity is deep, some form of zinc filling should be placed in first, to serve as a base for the proper shaping of the cavity. I have seen cases where the fillings have been put in, in the manner described, where the bottom was filled up with so-called soft foil, and then the surface finished with cohesive gold. The teeth wear down, after a while, and leave the non-cohesive gold, which does not stick together, and will peal off, and we have a filling that is not good. In the case of these fillings, put in 40 years ago, as Dr. Atkinson stated, the gold was made cohesive without the operator being aware of it; we find that most all of these fillings are upon the grinding surfaces, and upon the proximal surfaces, with the next tooth missing. Operators at that time did not attempt to perform the operations that are performed to-day.

In reference to the matter of its being easier to put in soft foil fillings, than with cohesive gold; that is a matter misunderstood; those who only occasionally put in cohesive fillings, cannot do it as well as others, who do it every day. I will state here that I know operators under 30 years of age, who are among the finest in the world to-day, and who do use cohesive gold; each of them to whom I refer has a large practice. How did they do it? They were not known before they commenced practice. They did it, because they did all their work well.

Dr. Noble.—I do not want Dr. Webb to think we put our cohesive gold *on;* we put it *in*, and if it is put there properly, there is no peel to it any more than his cohesive fillings. I have lost two valuable teeth, filled by good operators, with cohesive gold, and that started me on the other side. I desire to thank Dr. Webb, and have him feel my unbounded admiration for a man, who would do what he is doing by going around, and doing his best. If he can convince me that his practice is the correct one, I think I will fall right in and do it.

Dr. Donaldson.— I hope Dr. Webb will not fall into the error of supposing that we in Washington, do not believe we perform good operations, because we cannot get the money for them. I do think we perform good operations.

The President.—When we speak of non-cohesive foil, we mean there is something on the surface of it, making it non-cohesive. All gold, that is manufactured from pure gold is cohesive gold.

Dr. Noble.—That point was carried at the meeting of the American Dental Association, in Philadelphia, and was flatly denied by the Abbey manufacturers; men of our profession were taken to Mr. Abbey's manufactory to watch the whole process.

Dr. Winder.—Leslie said that all gold as it came from the manufacturers hand was cohesive, and stated that he dipped his gold into a solution, to make it non-cohesive.

Dr. Noble.—That was so, but then there was another man, I think it was Dr. Kingsbury, who said it was not so.

Dr. Atkinson.—Mr. Abbey has a peculiar form of Vellum which has red ink, of some kind, on it that may effect the gold, thus making it non-cohesive.

Dr. Hodgkin.—I can hardly think the conclusions arrived at are correct. Abbey's soft foil is not susceptible of being made cohesive by heating, while the others are to a great extent.

The President.—Has any one passed a piece of foil through an acid, and tried to make it cohesive? That would probably be a good test.

Dr. Webb.—If teeth are injured by malleting, it is the fault of the malleting. Malleting must be done properly, and I do know if it is done properly, a filling may be malleted for an hour, and the teeth not be sore afterwards. When I state that I often use from one to two, or three books a day, it will indicate to you that I have had some experience.

The President, stated that the Executive Committee had made arrangements for a clinic the following morning.

On motion of Dr. Winder it was ordered that the morning session be dispensed with, in order to allow the gentlemen more time for their clinics.

On motion of Dr. Winder the rules of the Society were suspended, and Drs. Webb, Baker, Underwood, and Reese were made honorary members.

On motion the subject of Operative Dentistry was passed, and the meeting adjourned.

